# Advancements in 3D In Vitro Models for Colorectal Cancer

**DOI:** 10.1002/advs.202405084

**Published:** 2024-07-04

**Authors:** Sara Vitale, Federica Calapà, Francesca Colonna, Francesca Luongo, Mauro Biffoni, Ruggero De Maria, Micol E. Fiori

**Affiliations:** ^1^ Department of Oncology and Molecular Medicine (OMM) Istituto Superiore di Sanità Viale Regina Elena 299 Rome 00161 Italy; ^2^ Dipartimento di Medicina e Chirurgia traslazionale Università Cattolica del Sacro Cuore Largo F. Vito 1 Rome Italy; ^3^ Fondazione Policlinico Universitario “A. Gemelli” – IRCCS Largo F. Vito 1 Rome Italy

**Keywords:** colorectal cancer, patient‐derived organoids, preclinical models for cancer, tumor microenvironment

## Abstract

The process of drug discovery and pre‐clinical testing is currently inefficient, expensive, and time‐consuming. Most importantly, the success rate is unsatisfactory, as only a small percentage of tested drugs are made available to oncological patients. This is largely due to the lack of reliable models that accurately predict drug efficacy and safety. Even animal models often fail to replicate human‐specific pathologies and human body's complexity. These factors, along with ethical concerns regarding animal use, urge the development of suitable human‐relevant, translational in vitro models.

## Graphical abstract

The tumor microenvironment is an interactive system of cancer and non‐cancer cells along with the extracellular matrix, playing a crucial role in cancer development and progression. 3D models and co‐culture techniques allow for patient‐derived tissue models incorporating a wide array TME components, providing a more comprehensive representation of colorectal‐cancer's complexity and personalized treatment response.

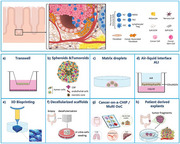

As tumors are highly complex, approaching the study of cancer cells’ biology without considering their interaction and co‐evolution with surrounding tissues is short‐sighted. Recent progress in isolating and cultivating primary cells in 3D models, and advancements in co‐culture techniques, enable the generation of patient‐derived tissue models that incorporate an increasing number of cell types from the tumor microenvironment. By integrating these models with state‐of‐the‐art technologies, it becomes possible to recreate crucial physical characteristics of tumors, such as matrix composition, stiffness, and mechanical stimuli resembling blood circulation. This review discusses the latest advancements in 3D in vitro models for colorectal cancer, and how they will support the personalization of treatments and the implementation of precision medicine approaches.

## 1 Tumor Microenvironment (TME)

Colorectal cancer is a heterogeneous disease, consisting of molecularly and clinically distinct subgroups, with differing prognosis and response to therapy. In addition to the heterogeneity observed between different CRC patients (referred to as intertumoral heterogeneity), involving genetic, histological and clinical variations, individual tumor masses encompass genetically and phenotypically different cancer cell populations, defined by a hierarchical organization, clonal diversity and interactions with the TME or stroma (referred to as intratumoral heterogeneity – ITH).^[^ ^1^, ^2^ ^]^ While genetic and epigenetic alterations are the main drivers of CRC initiation and progression, the interactions of tumor cells with the TME are also critical contributors. These interactions promote the activity of a subpopulation of cancer cells, namely Cancer Stem Cells (CSCs), responsible for tumor growth, survival, migration, and invasion.^[^ ^2^, ^3^, ^4^, ^5^ ^]^ The TME further sustains tumor development through metabolic reprogramming, providing nutrients and energy for cancer evolution, radio/chemo‐resistance and metastatic spread.^[^ ^6^ ^]^

The TME is an interactive milieu surrounding tumour cells, comprising both non‐cellular (extracellular matrix, ECM) and cellular elements. Here, we briefly describe the main components of both the matrix and the cells included in CRC TME.

The ECM has a “dynamic” nature, which promotes the renewal and repair of the microenvironment, and the loss of the correct ECM architecture is considered a hallmark of solid tumors. The ECM is composed of collagen, laminins, fibronectin, proteoglycans and hyaluronans. Collagen, the primary constituent of ECM, is highly expressed in CRC patients where elevated matrix density, stiffness, and fiber alignment have been associated with tumorigenesis, epithelial‐mesenchymal transition (EMT), invasion and unfavourable outcome.^[^ ^7^ ^]^ Several studies comparing healthy colon and CRC reported a progressive ECM re‐arrangement, characterized by a different biomolecular composition, increased collagen deposition and altered orientation, with linearized fibrils organized in tight bundles which substitute the random network of relaxed fibrils observed in the healthy ECM.^[^ ^8^, ^9^ ^]^ Furthermore, it was demonstrated that the stromal ECM of premalignant tissues undergoes early changes, preceding the appearance of tumor, suggesting that the mechanical pre‐strain of the ECM might predispose to cancer onset.^[^ ^8^, ^10^ ^]^ Moreover, interesting studies have shown a correlation between tensile forces in the ECM and the metastatic potential of CRC.^[^ ^11^ ^]^

A variety of cellular subtypes compose the TME, including endothelial cells, immune‐inflammatory cells, mesenchymal cells, neuro‐endocrine cells, adipose cells, and cancer‐associated fibroblasts (CAFs).^[^ ^3^ ^]^ Among the main components of TME, CAFs are key drivers in the formation of a permissive niche as they contribute to the deposition of ECM associated with tumor fibrosis and secrete paracrine factors (cytokines, EVs, metabolites) promoting cell growth, survival and migration, influencing metabolic and immune reprogramming of the TME, with an impact on response and resistance to therapy.^[^ ^12^, ^13^ ^]^ Moreover, fibroblast‐induced paracrine pathways can influence cancer cell plasticity, switching the balance toward stemness in CRC cells.^[^ ^14^ ^]^

The balance between effector and tolerogenic immune responses regulates tumor onset and progression and strongly impacts the therapeutic efficacy of available regimens. In principle, the immune system represents a potent defense mechanism against cancer development. However, aberrant cells evolve complex mechanisms to avoid immune recognition and trigger an immunosuppressive TME.^[^ ^15^ ^]^ Of note, the quantification of T cell infiltration (CD3^+^, CD4^+^ and CD8^+^) at the tumor core and invasive margin, named ImmuneScore, provides a superior prognostic power compared to TNM staging, correlating with recurrence, overall survival (OS), and disease‐free survival (DFS) in CRC patients, including metastatic subjects.^[^ ^16^ ^]^ Various strategies have been proposed to reverse the immunosuppressive TME and restore the natural host immune response to eradicate the tumor, as monoclonal antibodies targeting key immune checkpoints and engineered T‐lymphocytes with a chimeric antigen receptor (CAR‐T cells).^[^ ^17^, ^18^, ^19^ ^]^

**Figure 1:**
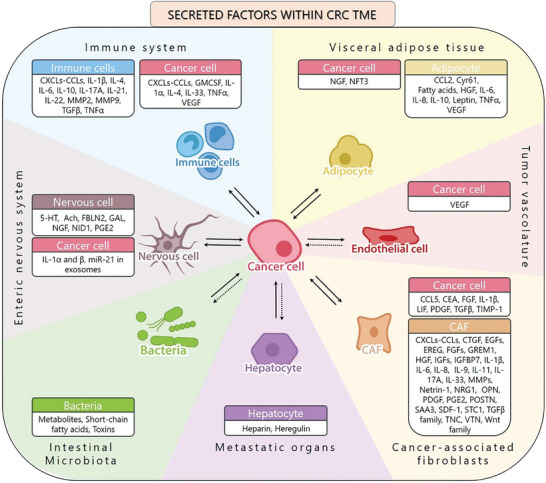
Crosstalk between CRC cells and different components of the TME. Schematic representation of the reciprocal communication network of colorectal cancer cells with the cell types that compose the tumor niche. Cells communicate mainly through soluble factors such as cytokines and chemokines, metabolites and extracellular vesicles, influencing tumor growth, immune responses, angiogenesis, and therapeutic outocomes in colorectal cancer. In each box are indicated the main soluble factors secreted by each cell type, that trigger pro‐tumoral pathways. Black arrows indicate direct communication, while dashed arrows signify indirect communication, not mediated by secreted factors. Among the principal components of the TME, cancer‐associated fibroblasts (CAFs) play a role in supporting colorectal cancer growth. CAFs and cancer cells exert a mutual influence by the indicated soluble cues (from CAFs to cancer cells,^[^ ^20^, ^21^, ^22^, ^23^, ^24^, ^25^, ^26^, ^27^, ^28^, ^29^, ^30^, ^31^, ^32^, ^33^, ^34^, ^35^, ^36^, ^37^, ^38^, ^39^ ^]^ from cancer cells to CAFs.^[^ ^21^, ^24^, ^36^, ^40^, ^41^, ^42^, ^43^ ^]^ Tumors require a blood supply for nutrients and oxygen. Blood vessels within the TME, known as tumor vasculature, can be abnormal, leaky, and disorganized. Cancer cells stimulate endothelial cells’ proliferation and neovascularization by releasing VEGF in the TME.^[^ ^44^ ^]^ The presence of cancer cells can trigger visceral adipose tissue to adopt an inflammatory pro‐tumoral phenotype,^[^ ^46^ ^]^ which in turn, produces molecules and metabolites that sustain tumor outgrowth.^[^ ^45^, ^46^, ^47^, ^48^, ^49^, ^50^, ^51^ ^]^ The immune system, namely T cells, B cells, natural killer (NK) cells, macrophages, and dendritic cells, infiltrate the TME. They interact with cancer cells and can have both pro‐tumor and anti‐tumor effects. Here we reported secreted factors produced by immune cells that sustain pro‐tumoral adaptation of cancer cells,^[^ ^52^, ^53^, ^54^, ^55^, ^56^ ^]^ and those secreted by cancer cells that induce a pro‐tumoral phenotype in immune cells.^[^ ^55^, ^56^, ^57^, ^58^, ^59^, ^60^, ^61^, ^62^ ^]^ The enteric nervous system has a pivotal role in physiological intestine functions. When properly stimulated by cancer cells,^[^ ^63^, ^66^ ^]^ it can sustain colorectal cancer stem cells self‐renewal and tumorigenesis, proliferation and metastatic spread.^[^ ^63^, ^64^, ^65^, ^66^, ^67^, ^68^, ^69^ ^]^ The intestinal microbiota can strongly impact tumor behaviour, by releasing in the TME metabolites and signaling molecules,^[^ ^70^, ^71^ ^]^ causing local inflammation or genetic damage of colonic epithelial cells and subsequent oncogenic transformation. Cells resident in metastatic sites communicate with cancer cells and support their homing and growth by secreting specific factors within the niche. Here, we represented hepatocytes and their secreted factors as the more abundant cell type in the liver, the most frequent metastatic site for CRC.^[^ ^72^, ^73^ ^]^

It seems now clear that tumor cells rely on the network of reciprocal interactions within the TME and that cancer features are strongly affected by this crosstalk. Paracrine communication within TME relies on cell‐cell physical interaction and on the production of soluble factors (for detailed information see **Figure** **1**).^[^ ^20^, ^21^, ^22^, ^23^, ^24^, ^25^, ^26^, ^27^, ^28^, ^29^, ^30^, ^31^, ^32^, ^33^, ^34^, ^35^, ^36^, ^37^, ^38^, ^39^, ^40^, ^41^, ^42^, ^43^, ^44^, ^45^, ^46^, ^47^, ^48^, ^49^, ^50^, ^51^, ^52^, ^53^, ^54^, ^55^, ^56^, ^57^, ^58^, ^59^, ^60^, ^61^, ^62^, ^63^, ^64^, ^65^, ^66^, ^67^, ^68^, ^69^, ^70^, ^71^, ^72^, ^73^ ^]^

Another form of paracrine communication is the secretion of extracellular vesicles (EVs).^[^ ^74^ ^]^ Tumor‐derived EVs carry various molecules such as nucleic acid (small RNAs, circular RNAs, lncRNAs, mRNAs and DNA),^[^ ^75^ ^]^ cytokines, chemokines and angiogenic factors. These molecules can induce matrix reprogramming and influence tumor growth, vascularization, EMT, immunoediting, migration, invasion, metastasis, and drug resistance.^[^ ^74^, ^76^, ^77^ ^]^ An interesting application of recent discoveries about EVs, is the development of a microfluidic chip with a porous structure for EVs capture and enrichment, allowing for specific antigen recognition. Coupled with a quantum dot detection method and machine learning intelligence analysis system, this chip was successfully applied as a diagnostic tool for CRC.^[^ ^78^ ^]^

A growing interest in the colonic microbiota as an active component of the tumor microenvironment (TME).^[^ ^79^ ^]^ has revealed that CRC patients present a pathological imbalance (dysbiosis) of the gut microbiome.^[^ ^80^ ^]^ This has led to the identification of specific microorganisms (i.e., pks+ Escherichia coli or Fusobacterium nucleatum) enriched in the CRC TME compared to the normal counterparts, which can contribute to cancer evolution and progression.^[^ ^71^ ^]^ Bacteria could affect the CR microenvironment through direct and indirect mechanisms that include metabolite secretion (i.e., short‐chain fatty acids, genotoxins, extracellular superoxide), epithelial signalling pathways dysregulation, and modulation of the immune response toward a proinflammatory protumoral state.^[^ ^70^, ^71^, ^81^, ^82^ ^]^

Although conventional monolayer cell culture systems have strongly contributed to our understanding of tumor biology, several studies have demonstrated that 3D models more accurately reflect the in vivo tumor condition in terms of cell connectivity, polarity, tissue architecture, gene expression profiles, protein expression, phosphorylation levels and the responses to treatment.^[^ ^83^, ^84^ ^]^

In recent years, the introduction of 3D culture systems coupled with the availability of primary cells, had a revolutionary impact on cancer studies. These systems have the unique capability to preserve genotypic and phenotypic traits of the original tumor and individual patient characteristics, enabling molecular characterization, drug screening, and the development of personalized approaches. However, commonly used 3D cultures do not fully reproduce the complex diversity of TME composition in CRC. They lack a complete multicellular representation, including stromal, immune, adipose and vascular cells, as well as musculature, nerves and microbiome, which are crucial for normal intestinal physiology.

**Figure 2:**
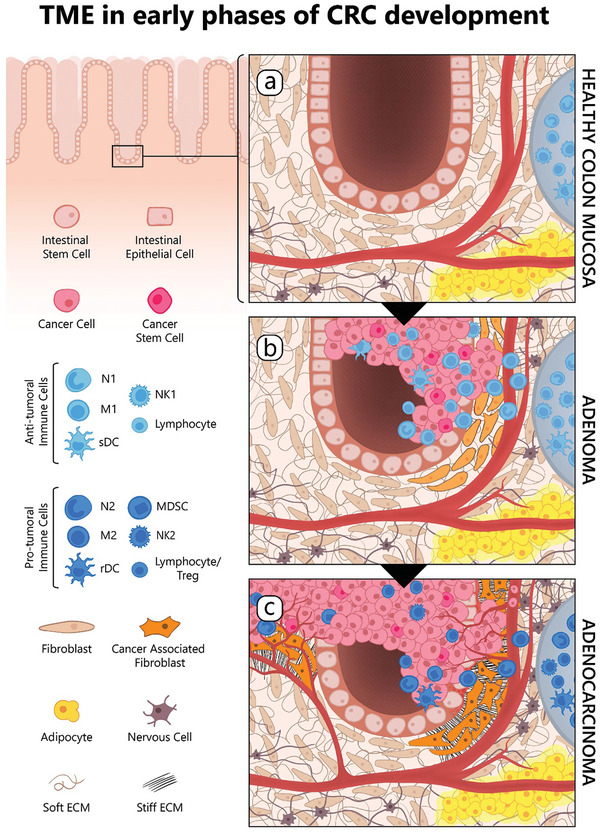
Co‐evolution of colorectal cancer and its tumor microenvironment. Schematic of CRC development and progression illustrates the cellular composition and the ECM organization of healthy colon mucosa and its modifications during CRC evolution. The legend on the left provides a comprehensive list of the key cellular players involved in this process. a) HEALTHY COLON MUCOSA‐Intestinal crypt homeostasis relies on intestinal stem cells located at the bottom of the crypt, and differentiated cells, such as the enterocytes, goblet, tuft, enteroendocrine and Paneth cells (indicated as “intestinal epithelial cells”). In healthy colon tissue, the microenvironment comprises quiescent intestinal fibroblasts surrounded by a soft extracellular matrix (“soft ECM”), alongside a network of blood vessels, nerve cells from the mesenteric plexus, gut‐associated lymphoid tissue organized into Peyer's Plaques (depicted as grey structures on the right) and visceral adipose tissue. b) ADENOMA‐During the early phases of CRC progression, the immune system promotes immunosurveillance, an inflammatory response aimed to control tumor proliferation through both innate (neutrophil N1, macrophage M1, stimulatory dendritic cell – “sDC” ‐, natural killer NK1), and adaptive immune cells (B and T lymphocytes). Immune cells mainly implicated in this phase are shown in the left legend (middle panel, light blue). In this context, quiescent fibroblasts (light orange) become reversibly activated, proliferate and support the inflammatory response. Cancer cells and cancer stem cells are indicated in pink and dark pink, respectively. c) ADENOCARCINOMA‐Through adaptive mechanisms and the accumulation of genomic mutations, cancer cells gain the capability to evade immunosurveillance (immunoescape) and hijack the immune cells to promote their own proliferation. Cancer cells may inhibit the function of immune cells or promote the recruitment of immunosuppressive cells to the tumor. This step is characterized by an immunological switch toward a more tolerogenic and immunosuppressive phenotype. Immune cells mainly implicated in this phase are shown in the left legend (lower panel, dark blue), neutrophil N2, macrophage M2, regulatory/tolerogenic dendritic cell (rDC), natural killer NK2, myeloid‐derived suppressor cell (MDSC), lymphocytes and T regulatory cells (Treg). Moreover, fibroblasts activated during the inflammatory phase, acquire irreversible features and secrete cytokines that support tumor growth and extracellular matrix proteins. ECM surrounding the tumor is characterized by high stiffness due to the composition and spatial organization of collagen fibrils, linearized into tight bundles (“stiff ECM”), that substitute the random network of relaxed fibrils observed in the healthy ECM (“soft ECM”). Tumor cells also induce proliferation of endothelial cells, leading to neoangiogenesis. Adipose and nerve cells support tumor growth by secreting specific factors. As tumor progression continues, cancer cells may acquire migratory capabilities and enter the bloodstream to colonize distant organs.

To address this limitation and reproduce the specific architecture and cellular network of CRC microenvironment in vitro, a growing number of 3D co‐culture models have been developed. These systems allowed to investigate the role of specific cell types in the TME and their contribution to tumor initiation, progression, and metastasis. **Figure** **2** describes TME adaptations during early phases of CRC development, highlighting the involvement of several cell types in supporting cancer cells’ proliferation.

Moreover, the introduction of single‐cell transcriptomics (single‐cell RNA sequencing – scRNA‐seq) has led to the identification of distinct cellular sub‐types within the TME, that contribute to tumor onset and development.^[^ ^85^, ^86^, ^87^ ^]^ This approach provides an unprecedented tool for dissecting intratumoral heterogeneity, highlighting the challenges involved in fully reconstructing the complex TME in vitro.

In the following sections, we will discuss recent advancements in 3D tumor co‐culture systems, with an emphasis on their ability to replicate the characteristics of CRC patients and the intricate networks within the microenvironment.

## 2 Technologies for In Vitro Modeling of CRC

**Figure 3:**
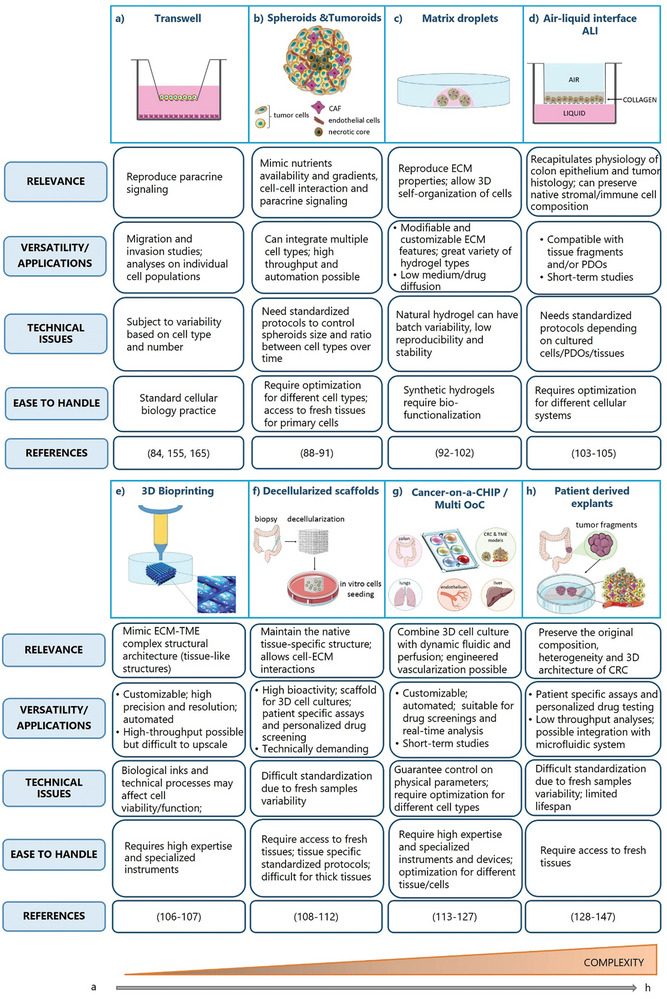
Strategies to mimic CRC in vitro. 3D in vitro technologies to reproduce CRC TME and cell‐cell interactions are shown. For each system, relevance, versatility/applications (including advantages and limitations), technical issues, handling and related references are described in the boxes. The techniques are arranged from the least to the most complex (orange triangle below). a) Transwell is an indirect co‐culture technique that reproduces paracrine communication between cells that share the same medium but are physically separated by a permeable membrane. b) Spheroids and tumoroids are 3D cell aggregates that allow the self‐organization of different cell types (cancer cell and TME cells) and mimic some features of a solid tumor mass, such as detachment from a substrate, nutrients availability and gradients, cell‐cell interaction and paracrine signaling. c) Matrix droplets allow to reproduce the ECM complexity using different types of 3D matrices (hydrogel, Matrigel, collagen). This system can be combined with other technologies such as bioprinting or microfluidic devices (e; g). d) ALI is a cell culture system in which cells are seeded on a semi‐permeable membrane, either embedded within a matrix (as in c) or in liquid medium. Their basal membrane is in contact with culture medium and their apical surface is exposed to the air. e) 3D bioprinting is a technique that allows the fabrication of biological constructs through the precise deposition and localization of multiple layers of cells in supporting biopolymers. f) Decellularized scaffolds are natural ECMs that maintain the biochemical and ultrastructural properties of the tissue of origin. The process of decellularization enables complete cell removal while maintaining tissue architecture and ECM components. g) Cancer‐on‐a‐CHIP/Multi OoC are microfluidic devices designed to mimic in vitro the physiological conditions of tumors including cellular interactions, biochemical and niche factors gradients, tissue barriers, vascular perfusion and mechanical forces. h) Patient‐derived explants (PDEs) allow the culture of freshly resected tumor fragments or slices, while preserving the native 3D tissue architecture and cellular composition (tumor cells, stromal cell, TILs). Part of the figure is modified from Servier Medical Art (http://smart.servier.com/), licensed under a Creative Common Attribution 3.0 Generic License.

In this section, we describe the available technical approaches to study in vitro CRC and TME interactions, highlighting the advantages and limitations of each technology (For a schematic overview see **Figure** **3**).

### 2.1 Transwell

The use of transwells provides a simple mean to culture different cell types in the same environment, the cells share the medium and communicate with each other by soluble cues, while they are physically separated by a membrane. This allows for downstream analyses of individual cell types to unravel at the molecular level the reciprocal influence of the selected cell population and cancer cells.

### 2.2 Spheroids and Tumoroids

In the latest decades, the development of 3D cultures expanded the potential of in vitro models, allowing a more accurate reproduction of tumor cell biology. Defined as scaffold‐free, floating aggregates of cells, spheroids, are a superior model compared to cancer cells grown in 2D monolayers. They mimice cancer cell features in terms of detachment from a substrate, nutrients availability and gradients, cell‐cell interaction, and paracrine signaling. Spheroids can be derived from monolayer cultures by forcing their anchorage‐independent growth as free‐floating or suspended aggregates using various methods (low attachment plastic ware, centrifugation in U‐shaped wells, hanging‐drop technology, magnetic forces etc). However, the availability of primary cancer cells directly cultured as spheroids, enriched in CSCs, provides even more robust 3D models with significant advantages.^[^ ^88^, ^89^, ^90^ ^]^ The integration of 3D cultures with other components of the tumor niche (hereafter named “tumoroids”) and the development of new technologies are leading to increasingly reliable in vitro models. Interestingly, co‐culture systems of gut microbiota and CRC spheroids have been developed.^[^ ^91^ ^]^ for a more comprehensive reproduction of the gut mucosa microenvironment and its cellular composition.

### 2.3 Matrix Droplets: Hydrogel‐Based Cultures

Scaffold‐based 3D cultures utilize matrix droplets, allowing for 3D self‐organization of cells in an ECM‐like environment. Organoids are the main application of this technology, they are embedded in matrices of animal origin and supplemented with specific growth factors. Organoids can be generated from different tissues of origin, including cancer.^[^ ^92^, ^93^, ^94^, ^95^, ^96^, ^97^, ^98^, ^99^ ^]^ Matrix droplets are exploited also for co‐cultures of cancer cells and specific components of TME, enabling them to “build” 3D structures resembling the reciprocal spatial organization observed in tumors. Here, we briefly discuss the main features of available matrices. Hydrogels are insoluble reticulated structures of crosslinked hydrophilic polymer chains, they are easy to handle, extremely versatile and suitable for ECM‐mimetic platforms. Hydrogels represent adaptable tools for creating scaffolds or matrices used for cell culture and tissue engineering. Based on their source, hydrogels can be classified in natural, synthetic and hybrid.

Natural hydrogels derive from biological sources such as proteins and polysaccharides. They include collagen, chitosan, fibrin, Matrigel and decellularized extracellular matrix. They are intrinsically biocompatible, biodegradable and promote multiple cellular functions due to their content in growth factors and integrin binding sites. As a drawback, they have a complex composition and limited stability, and they may have batch‐to‐batch variability that can impact on the reproducibility of experimental settings.

Conversely, synthetic hydrogels are composed of synthetic polymers such as polyacrylamide (PAM), polyvinyl alcohol (PVA) or polyethylene glycol (PEG). They offer a greater control of biochemical and mechanical properties and high reproducibility but are biologically inert, lack endogenous factors, and require bio‐functionalization.^[^ ^100^, ^101^ ^]^

Hydrogels are sometimes prepared in a hybrid mode, to match their respective advantages and mimic microenvironment complexity. Further, they can be used as stand‐alone 3D matrices or combined with other technologies such as microfluidic devices, bio‐printing and cellular microarrays.^[^ ^101^, ^102^ ^]^

### 2.4 Air‐Liquid Interface

The air‐liquid interface (ALI) culture system allows cell propagation upon exposure to both liquid medium/matrix and air, thus mimicking the physiological condition of some epithelia, such as lung and colon. One interesting application of ALI systems foresees cancer cells or mechanically dissociated tumor intestinal fragments layered on a permeable insert pre‐coated with a collagen matrix, creating two compartments where cells exposed to air are on the apical surface, while the basolateral layers are submerged in a liquid medium, mimicking the environment of intestinal tissues.^[^ ^103^ ^]^

Tumor ALI‐organoids composed by both epithelial and stromal cell components, closely recapitulate epithelial structures of the original tumor and show resistance to chemotherapy, representing a useful system for drug testing in the TME.^[^ ^104^ ^]^ Neal and colleagues established ALI cultures of patient‐derived organoid (PDO) from 100 primary and metastatic patients’ tumors, representing 19 tissue sites and 28 disease subtypes.^[^ ^105^ ^]^ These organoids could be expanded, serially passaged, cryopreserved, and xenografted into immunocompromised mice and re‐derived as organoids. ALI PDOs recapitulated the parental tumor histology, preserving tumor architecture and native stromal/immune cell composition for up to 2‐months. Genetic features of the original tumor were also maintained. Of note, in the ALI PDO system, native infiltrating immune cell populations were also preserved, including CD3^+^ T cells expressing PD1, cytotoxic T cells, Th, B, NK, NKT cells and macrophages, thus comprehensively reproducing the tumor immune microenvironment (TIME). Moreover, tumor‐infiltrating lymphocytes (TILs) were functional and maintained the original TCR repertoire observed in patients, with a response to immune checkpoint inhibition (anti‐PD1 antibody Nivolumab) comparable to clinical data.^[^ ^105^ ^]^

Although further studies will be required to obtain a long‐term PDO preservation of immune cell subsets, ALI PDO constitutes a promising tool for the development of personalized therapies.

### 2.5 Bioprinting

**Figure 4:**
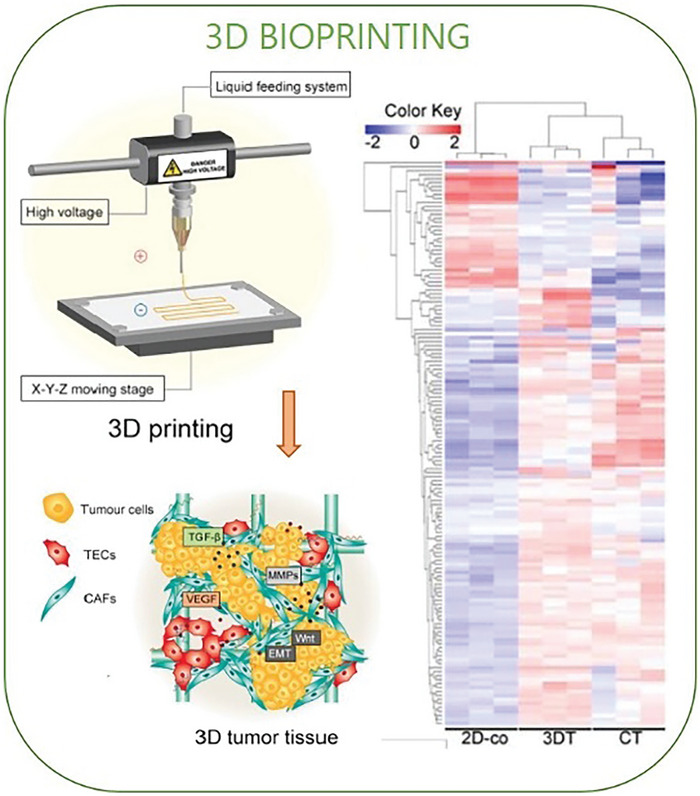
3D bioprinting. 3D bioprinting is an advanced technology that enables the creation of complex 3D biological structures that mimic the native tissue architecture, by precisely arranging multiple layers of cells and/or supporting biopolymers. The figure represents a schematic of a 3D bioprinting device (*left upper panel*) that was employed for the fabrication of a collagen‐based 3D scaffold, subsequently populated with tumor cells, cancer associated fibroblasts (CAFs) and tumor‐associated endothelial cells (TECs) (*left lower panel*). Adapted with permission.^[^ ^107^ ^]^ Copyright 2020, Ivyspring International Publisher. Transcriptomic analyses reveal that CRC avatars grown on 3D‐bioprinted scaffolds better reproduce gene expression of in vivo models, if compared to 2D cell‐cultures. This is shown in the *right panel* containing a clustered heatmap of 142 metabolic genes expressed in the 2D co‐cultured cells (2D‐co), 3D tumor tissue (3DT), and in vivo colorectal tumor (CT). Adapted with permission.^[^ ^107^ ^]^ Copyright 2020, Ivyspring International Publisher.

By integrating modern approaches with computer‐aided design technologies, the 3D bioprinting process controls the microarchitecture of complex co‐cultures in vitro, through the precise deposition and localization of multiple layers of cells and supporting biopolymers (bioinks). Due to the availability of a wide range of printing materials and manufacturing processes, this technology provides a flexible solution to reproduce the physiological niche through the addition of cells, bioactive molecules, and biomaterials for tissue engineering, disease modeling and drug testing.^[^ ^106^ ^]^ For instance, Chen H. and colleagues observed that a co‐culture of CRC cells, CAFs and tumor‐associated endothelial cells (TECs) in a 3D‐printed scaffold promotes adhesion, pre‐vascularization, matrix remodeling, network structures formation, stemness, and proliferation. Furthermore, this in vitro 3D tumor tissue displayed metabolic signals, malignant transformation and high drug resistance similar to that observed in mouse xenografts in vivo (**Figure** **4**).^[^ ^107^ ^]^

3D bioprinting holds the potential for studying cancer growth and progression, anti‐tumoral drug response and personalized patient treatment. This approach guarantees indeed high precision and resolution, homogeneous distribution of cells, high reproducibility of ECM‐TME properties and complex structural architecture. However, it is less amenable to high‐throughput screenings and can be technically challenging as biological inks or the manipulation and procedures may affect cell viability and function.

### 2.6 Decellularized Scaffolds (dECMs)

**Figure 5:**
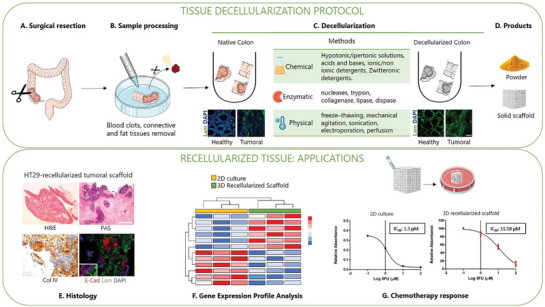
Preparation and Applications of dECM. The procedure to obtain dECM is described in the upper box, comprising the main steps of available protocols A–D). A) Following surgical resection, fresh colon tissues are promptly transferred to the collection medium and processed. B) In a petri dish, fat and blood clots are removed from tissues and rinsed in sterile PBS. C) Samples are cut and decellularization is achieved through chemical, enzymatic and/or physical treatments.^[^ ^108^, ^109^, ^112^ ^]^ Upon completion of the process, tissue turns translucent and DNA content is significantly reduced. Immunofluorescence images in panel C show the loss of cellular content in both decellularized healthy mucosa and CRC samples (DAPI staining‐blue) while preserving the matrix structure (Laminin staining‐green). Adapted with permission.^[^ ^111^ ^]^ Copyright 2020, MDPI. D) Finally, the decellularized scaffold can be utilized for recellularization or pulverized into a fine powder and used as hydrogel in 3D co‐culture, microfluidic devices and bioprinting.^[^ ^102^, ^110^, ^112^ ^]^ The lower box shows the main applications of recellularized scaffolds. Recellularization of dECM derived from healthy mucosa, CRC or metastatic sites enables the creation of models for tumor progression and metastasis as well as in vitro platforms for drug screening or gene expression profiling.^[^ ^109^, ^110^, ^111^ ^]^ E) Example of histological characterization of CRC‐derived scaffold recellularized with HT‐29 cells, hematoxylin and eosin (H&E); periodic acid‐Shiff (PAS); collagen IV staining (Col IV); IF staining with epithelial marker E‐Cadherin (E‐Cad), basement membrane marker Laminin (Lam) and DAPI [adapted from reference 111]. G) Example of dECM application in chemotherapy response studies, IC50 values indicate higher resistance to 5FU in HT29 cultured within 3D scaffolds compared to 2D monolayers. Adapted with permission.^[^ ^111^ ^]^ Copyright 2020, MDPI. Part of the figure is modified from Servier Medical Art (http://smart.servier.com/), licensed under a Creative Common Attribution 3.0 Generic License.

Tissue decellularization provides a novel approach to exploit natural tumor ECM. Through specific detergent and enzymatic treatments, this procedure enables complete cell removal, maintaining tissue architecture and ECM components, and producing scaffolds that recapitulate the histological, biochemical and ultrastructural properties of the tissue of origin (**Figure** **5**).^[^ ^108^ ^]^ In 2016, Shuler's lab generated a physiologically active *ex vivo* model of the human colon by decellularizing healthy colon mucosa that retained the tissue's geometry and preserved the ECM, the vascular network and an intact muscularis mucosa. By reseeding primary colonic epithelial cells, endothelial cells and myofibroblasts, they were able to replicate colon features in vitro and exploited this model to mimic CRC progression by introducing specific mutations and modulating TGFβ levels. Further, they performed a genetic screen with this model system, to identify new driver genes in CRC.^[^ ^109^ ^]^

D'Angelo and colleagues established a 3D model of colorectal liver metastasis (CRLM) and matched CRC, using decellularized patient‐derived liver matrix, recellularized with CRC cell lines. CRLM scaffolds supported CRC cell adhesion and influenced gene expression, proliferation, apoptosis, migration and drug response.^[^ ^110^ ^]^ In a subsequent study, surgically resected CRC tissue and adjacent healthy colon mucosa were decellularized to establish a 3D preclinical platform for drug screening. The authors demonstrated that the tested drug diffused through 3D CRC scaffolds and co‐localized with the cell nuclei. Moreover, the response to 5‐Fluorouracil (5‐FU) treatment observed in the in vitro CRC 3D model was comparable to the effect registered in vivo (xenotransplanted zebrafish).^[^ ^111^ ^]^ In a similar approach, decellularized mouse organs were used to engineer cancer metastasis in a tissue‐specific manner. CRC cells cultured in vitro on decellularized liver or lung scaffolds formed 3D colonies resembling patient's metastases. Moreover, compared to conventional culture substrates, cells grown on dECMs presented differentiated therapy‐response, reflecting the organ of origin of the dECM on which they were cultured and retained their organ‐specific tropism when injected into mice.^[^ ^112^ ^]^

### 2.7 Cancer on a Chip (CoC)

An Organ‐on‐a‐chip (OoC) is a microfluidic cell culture device designed to replicate in vitro the biology of human organs, including cellular composition, spatial organization, physiological functions, and mechanical properties. It typically consists of one or more transparent microfluidic chambers adapted for the growth of living cells (primary cells, cell lines, organoids, tissue sections/explant). These cells can be subjected to fluid flow and mechanical forces that mimic human body physiological conditions. Several OoCs have been established that successfully replicate healthy organs.^[^ ^113^, ^114^, ^115^ ^]^ In this exciting field, livers‐on‐chip stand out as important preclinical tools to predict drug toxicity in humans.^[^ ^116^ ^]^

**Figure 6:**
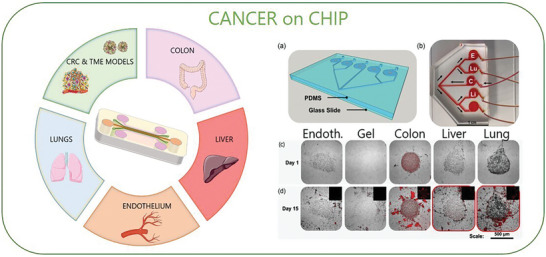
Cancer on a chip (CoC). Schematic of multi‐organ on chip (Multi‐OoC) for CRC modelling. By combining multi‐organ systems and 3D cell culture, this technology enables the connection of CRC cells and TME models with multiple organs and tissues in a closed‐loop system. Moreover, this platform offers a powerful tool for mimicking cancer metastasis (metastasis‐on‐a‐chip). For example, it integrates CRC cells with liver, lung, and endothelial cells to reproduce the tropism of CRC cells during spreading to distant organs. Adapted with permission.^[^ ^125^ ^]^ Copyright 2018, Wiley Periodicals, Inc. Panel A) shows an example of a metastasis‐on‐a‐chip device consisting of a main microfluidic chamber seeded with CRC cells, connected to multiple downstream chambers in which endothelial (E), lung (Lu), liver (Li), and gel control constructs are housed B). Under recirculating fluid flow, the movement of cells can be tracked via fluorescent imaging (RFP+ cells) C) from the primary site to 4 downstream potential sites of metastasis, reproducing CRC cells spread and tropism for the liver and lungs. Adapted with permission.^[^ ^125^ ^]^ Copyright 2018, Wiley Periodicals, Inc. Part of the figure is modified from Servier Medical Art (http://smart.servier.com/), licensed under a Creative Common Attribution 3.0 Generic License.

This technology has been successfully applied to cancer research, resulting in the generation of so‐called “Cancer on a chip” (CoC) models (**Figure** **6**). This term refers to different kind of devices that were developed to mimic in vitro the biological and physical properties of tumors, including biochemical gradients, niche factors, tissue barriers, mechanical forces, and vascular perfusion, combining the advantages of microfluidic and 3D cell culture technology. Multiple cell types can be cultured in CoC compartments and injectable hydrogel or dECM are often added to support cell growth and migration, while the microfluidic system perfuses cell culture medium through or across tissue structures providing oxygen and nutrients diffusion as well as biomechanical stimuli.^[^ ^117^, ^118^, ^119^ ^]^ Some studies have shown that fluidic systems offer several advantages over static cultures, particularly when focusing on pharmacokinetic and drug delivery.^[^ ^120^ ^]^

CoC platforms have proven useful for high‐throughput drug screening applications and to investigate several aspects of metastatic progression. An interesting study on CRC described a CoC consisting in a “tumor” core (CRC cell lines ‐HCT‐116) embedded in Matrigel in a circular chamber, supported by an adjacent “microvascular” network (human colonic microvascular endothelial cells–HCoMECs) able to invade the hydrogel core and reconstitute the beginning of the angiogenic sprouting process. This model allowed testing of drug‐loaded nanoparticles through a dynamic gradient and subsequent gene expression analyses in tumor cells following therapy.^[^ ^121^ ^]^ Fascinating studies were focused on reproducing the spread of CRC cells to the metastatic sites using CoC models. To this end, Sterlez and colleagues developed a CRC‐on‐chip, integrating multiple key cell types of the TME and physical forces mimicking peristalsis, to understand how the TME influences intravasation, an early step of metastasis, and the spread of primary CRC cells. The device consists of epithelial (Caco2, C2BBe1) and endothelial (HUVEC) compartments, separated by a porous membrane. CRC cells (HC116 or HT29) or PDOs were injected into the epithelial channel and monitored via on‐chip imaging and mass spectrometry‐based metabolomics. CRC cells intravasate from the epithelial to the endothelial compartment, with invasive proficiency reflecting the aggressive features of cancer cells. Moreover, CRC invasiveness was influenced by the presence of CAFs and by applied physical forces, revealing the fundamental role of TME components in the behavior of tumor cells.^[^ ^122^ ^]^

An intriguing branch in the field of CoC foresees their integration into multi‐organ systems (multi‐OoC), aiming at interfacing cancer cells with multiple organs/tissues in a closed loop. These models emerge as powerful predictors of metastatic progression and offer close‐to‐physiological conditions to study and interfere with cancer spread.^[^ ^123^ ^]^ In 2016, Skardal and colleagues developed a modular system consisting of 2 chambers connected in series, populated with cells from human colon epithelium (Int‐407 cells) or human hepatoma cells (HepG2), respectively, and a tuneable hydrogel. The fluidic device had a peristaltic pump that perfused growth medium in a close circuit. By seeding in the colonic epithelial chamber fluorescent CRC cells, they were able to monitor liver colonization to study the impact of ECM stiffness on cancer cell motility and spread, as well as test drug treatment efficacy in preventing metastatic diffusion.^[^ ^124^ ^]^ Two years later, an evolution of this system was published by the same author, who realized a multi‐organ perfusable system, connecting the main chamber populated by CRC cells (fluorescent HCT‐116) with four other chambers by a double branching microfluidic channel (see Figure 5 –NEW). In each chamber, a different organ was mimicked by seeding specific cell types in a UV‐cross‐linked hydrogel matrix in 3D (HepG2, human liver hepatoma cells, A549, human lung epithelial cells cancer cells, and HUVEC, human umbilical vascular endothelial cells). The fourth chamber contained only matrix, as a control. The authors were able to reproduce CRC cells tropism for liver and lungs, consistent with the clinical evolution in CRC patients.^[^ ^125^ ^]^ Another interesting application of multi‐OoC to the study of CRC, investigated the hepatic metabolism of anti‐cancer prodrugs, and their effect on CRC cells and multiple organs. Capecitabine (CAP) is a precursor drug that is converted by hepatic cells into 5‐FU, one of the most relevant chemotherapeutics in CRC line of treatment. Satoh and colleagues developed a platform featuring a liver and a CRC compartment, connected by a pneumatic‐pressure‐driven medium flux. They were able to monitor the cytotoxic effect of 5‐FU on cancer cells through the transformation of CAP into its active metabolite. This model was further implemented up to four organs (CRC, liver, healthy colon, and connective tissue).^[^ ^126^ ^]^

The challenges connected with these 3D models are still numerous, but the undisputed advantages they offer will prompt their clinical applicability.^[^ ^127^ ^]^

### 2.8 Patient‐Derived Explants

**Figure 7:**
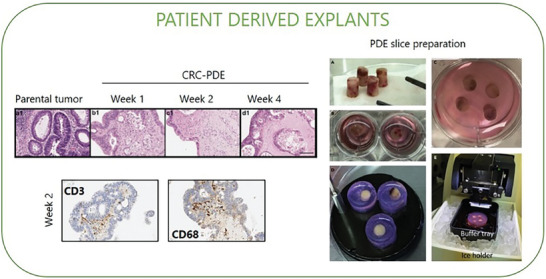
Patient‐derived explants (PDE). Explants can be generated by fragmentation (PDE) or slicing (tumor slice) of fresh tumor tissue. In the left panels, representative H&E images of colorectal parental tumour tissue and derived explants (CRC‐PDE) over 4 weeks of culture; representative immunohistochemical images of T lymphocytes (CD3‐positive) and macrophages (CD68‐positive) detection on the CRC‐derived explants at weeks 2 of culture. Adapted with permission.^[^ ^144^ ^]^ Copyright 2021, MDPI. In the right panels, an example of PDE slice preparation, tumor tissue cores are collected from fresh specimens A) and placed on ice in a storage solution; then tissue cores are embedded in agarose individually B) or grouped C) and adhered to specimen disc D). The disc is placed on ice in the buffer tray with a cutting solution for slicing E). Vibratome cuts cores into tumor slices with desired thickness and placed them in a dish with medium for culture. Tumor slices can undergo evaluation for treatment response, viability, histology, imaging, and multi‐omics analyses. Adapted with permission.^[^ ^131^ ^]^ Copyright 2021, Elsevier.

Current in vitro approaches implicate the dissociation of tumor samples and the use of specific culture conditions, which inevitably select some cell populations while don't support others. Although the above‐described recent technologies, such as organoids, microfluidics and new biomaterials offer the possibility to incorporate missing cell subsets in a 3D system, the reconstitution of the original tumoral composition is often incomplete. *Ex vivo* systems such as patient‐derived explants (PDEs) allow the culture of freshly resected fragments from human tumors (or normal adjacent tissue) with the preservation of native 3D tissue architecture and cellular components (i.e., stromal cell, tumor‐infiltrating lymphocyte).^[^ ^128^ ^]^ Explants can be generated by fragmentation (PDE) or slicing (tumor slice) of fresh tumor tissue using instruments such as vibratome or tissue chopper.^[^ ^129^, ^130^, ^131^ ^]^ Sample thickness may range from a few millimetres to several centimeters for PDE, and from 150 to 350 µm for slices, depending on the experimental setting (**Figure** **7**). Then explants can be maintained in culture using different protocols ranging from total submersion of tissue fragments/slices in media, with the support of a metal or plastic grid, to culturing on top of gelatin/collagen sponge scaffolds.^[^ ^128^, ^132^ ^]^

Specific cell composition, spatial profiling or drug responses in the PDE could be evaluated using hematoxylin and eosin,^[^ ^133^, ^134^ ^]^ immunohistochemistry,^[^ ^133^, ^134^, ^135^, ^136^ ^]^ immunofluorescence,^[^ ^135^ ^]^ mass spectrometry.^[^ ^137^ ^]^ or analyzing protein, DNA, RNA or metabolites from homogenized explants.^[^ ^137^ ^]^ PDE technology was successfully employed with different solid tumors, including breast,^[^ ^138^ ^]^ prostate,^[^ ^138^ ^]^ NSCLC,^[^ ^137^ ^]^ ovary,^[^ ^139^ ^]^ endometrium.^[^ ^139^ ^]^ and CRC.^[^ ^133^, ^135^, ^140^, ^141^, ^142^, ^143^ ^]^ A tumor explant model from advanced CRC patients with liver metastases showed the induction of tumor cell death and the inhibition of tumor‐promoting cytokines upon treatment with CCR5 inhibitor (maraviroc). Concordant results confirmed the therapeutic efficacy of maraviroc in patients with advanced‐stage metastatic CRC enrolled in a phase I clinical trial.^[^ ^135^ ^]^

PDEs represent a powerful tool for translational research and personalized medicine, explants are easy to obtain and relatively inexpensive (as compared to patient‐derived xenograft ‐PDX or organoids); they preserve tumor heterogeneity, allowing a rapid and comprehensive evaluation of drug and immunotherapeutic agents, targeting both tumor and stroma, with a high potential to be predictive of patient response in vivo. The short‐term duration of explant cultures, typically up to 72 h, hinders anticancer drugs testing to a restricted window of time.^[^ ^128^ ^]^ Limited life span of tissue explants is generally due to the lack of vascularisation, with consequent absence of oxygen/nutrients supply or waste removal.

Although explants have been reported to be more viable in short‐term culture, they can also grow for longer period while retaining properties of native tissue. In this regard, Mata et al. were able to maintain CRC‐PDE for over 4 weeks of culture using an agitation‐based platform, which improved oxygen and soluble factors diffusion.^[^ ^144^ ^]^ Moreover, the ability to integrate microfluidic technology with tissue explants/slices (referred tissue‐on‐chip) allows for precise control of factors like flow, nutrients, and waste removal within the surrounding microenvironment, enabling the monitoring of tissue responses and cell interactions. To this end, Yissachar N. and colleagues developed a 3D microfluidic organ culture for the gut, based on modifications of the ALI system. The organ culture features mouse‐excised intestinal segments connected to input and output ports, controlling respectively medium flow inside the lumen (with the delivery of molecules and microbes) and in the external medium chamber. Intestinal segments were semi‐submerged in media to replicate ALI culture and maintain tissue integrity. Although the tissue remained viable for several days, some loss of epithelial integrity was observed after 24 h, limiting the experiments to this time frame. This 3D microfluidic approach supports the growth of gut microbiota and reveals novel interactions among intestinal cells, the immune system, microbes, and nutrients.^[^ ^145^ ^]^

Overall, these approaches provide useful tools for the study of intact living samples within their autologous environment, with promising potential in fields like cancer biology, drug discovery, and personalized medicine for therapeutical responses.^[^ ^146^, ^147^ ^]^

## 3 Co‐Culture Systems

To reproduce cell‐cell interactions and spatial distribution within the TME of colorectal cancer, a large number of studies have elaborated various 3D co‐culture systems, exploiting the above‐described technologies. Different CRC cellular models have been challenged in 3D co‐cultures, spanning from commercial cell lines grown in 3D conditions to patient‐derived primary cells, mainly grown as spheroids or organoids. Regarding TME cellular components, immortalized cells derived from healthy tissues have been widely used to study how the interaction with transformed cells may impact their phenotype and function. Interestingly, the possibility to isolate and characterize primary cells of the TME from surgical CRC specimens allowed the development of more reliable models and paved the way for functional studies dissecting the role of distinct cellular subtypes within the CRC niche. Here, we provide some meaningful examples of co‐culture systems applications that shed light on CRC biology, with a special focus on CAFs and immune cells.

### 3.1 Double Cultures

Co‐culture platforms including cancer cells and a unique cellular component of the TME enable the simplification of the complex network of structural and functional interactions within tumors. This approach allows the study of direct communication between cancer cells and other cell types, leading to the identification of the main mediators of tumor‐niche crosstalk. Besides the use of transwells, another simple approach is the co‐culture of adherent cells (like fibroblasts) as monolayer with cancer cells grown in 3D as spheroids or organoids, embedded or not in matrix droplets.^[^ ^148^ ^]^ More and more sophisticated systems are currently employed to better mimic tumor composition and spatial organization, with the final goal to understand the contribution of single TME components to tumor development. Here, we will briefly describe the most relevant double culture approaches applied to the study of fibroblasts and immune cells.

#### 3.1.1 Dissecting the Role of CAFs: Cancer Cells and Fibroblasts 3D Co‐Cultures

##### Structural Role of CAFs

The use of 3D co‐cultures led to a better understanding of the structural role of CAFs, exerted both by molding ECM composition, organization and stiffness and through their physical interaction with cancer cells. By leveraging a 3D collagen gel culture model, it was demonstrated that invasive cancer cells migrate on elongated protrusions of CAFs, both individually and as cell clusters (collective invasion). Cancer cells can invade the collagen matrix mainly through the direct interaction of cellular integrin‐α5β1 and the fibronectin fibrils assembled on fibroblasts, showing how CAFs can indeed mechanically guide migration and matrix invasion.^[^ ^149^, ^150^ ^]^

Increasing evidence shows that matrix stiffness has profound effects on tumor growth and metastasis.^[^ ^151^ ^]^ By modulating matrix concentrations and composition in 3D co‐culture systems, it was possible to mimic in vitro how stiffness gradients affect cell migration and other cancer cell hallmarks.^[^ ^152^, ^153^ ^]^ Besides biochemical cues, matrix composition and stiffness can be remodeled by the mechanical traction exerted by and between neighboring cells. This reciprocal and dynamic influence between ECM and cells has a strong impact on tumorigenicity and cancer evolution. Given the importance of the mechanical interactions within the tumor mass, 3D co‐cultures have been exploited to reach a deeper understanding of this process. A high‐resolution micro‐fabricated sensor, integrated with a collagen 3D co‐culture system, was developed to measure tissue stiffness changes within the tumor model in vitro. By this method, Emon and colleagues showed that CAFs induce ECM stiffness that increased by three times in just 24 h of co‐culture with CRC cells.^[^ ^154^ ^]^

Interestingly, the rearrangements of the ECM network mediated by CAFs can influence also the physical properties of the basal membrane (BM) making it permissive to cancer cell invasion. This can be mimicked by a co‐culture system where cancer cells are seeded on one side of a decellularized mouse mesentery (as a model of BM) and CAFs are embedded in type I collagen on the other side. CAFs enhance the invasion capacity of cancer cells and reduce BM stiffness, through an MMP‐independent but contractility‐dependent mechanism.^[^ ^155^ ^]^

##### Paracrine Role of CAFs

Besides impacting tumor cell migration, invasion and ECM remodeling, the introduction of fibroblasts into the cultures induces metabolic changes, promotes EMT and drug resistance mainly through paracrine communication with surrounding cells.

CAFs and cancer cells within the tumor niche co‐evolve through a sophisticated reciprocal dialogue driven by soluble mediators. Using 2D and 3D co‐colture systems, Mosa and colleagues observed that colon cancer progression is influenced by CAFs that can adopt distinct phenotypes depending on the Wnt signaling level. Low stromal Wnt level supports an inflammatory‐like CAF subtype (iCAF), which induces a significant upregulation of EMT markers and invasiveness in tumor organoids; on the contrary, the contractile myofibroblastic CAFs (myCAF) subtype is induced by high levels of Wnt and promotes tumor growth.^[^ ^156^ ^]^

In the past few decades, the role of CAFs in resistance to therapies for gastrointestinal cancers has emerged. The co‐culture model stood up as a biologically relevant setting to study the mechanisms behind resistance.^[^ ^157^ ^]^ and to explore the therapeutic potential of reducing/targeting CAF populations and ECM deposition in the colorectal microenvironment.^[^ ^158^, ^159^, ^160^ ^]^

Lately, co‐cultures of CRC PDOs and CAFs, compared to PDO monocultures, were able to recapitulate the gene expression patterns and pathways associated with tumor‐stroma crosstalk found in CRC patient tissues. Using single cell RNA Seq (scRNAseq), Strating and colleagues identified three different clusters of cancer cells ad three CAFs subtypes. They show that co‐culture increases a cancer cell cluster characterized by partial EMT, similar to what is observed in consensus molecular subtype 4 (CMS4) colon cancer. At the same time, co‐cultured CAFs resemble a specific subpopulation of glycolytic myofibroblasts that is enriched in CMS1 and CMS4.^[^ ^161^ ^]^ Other studies showed that CAFs are able to support organoid growth without the addition of specific niche factors used in conventional organoid medium.^[^ ^162^, ^163^ ^]^ In this co‐culture settings, organoids and CAFs displayed a broad cellular heterogeneity and closely resemble primary tumor in terms of histology, biophysical interactions, gene expression and molecular subtypes.

This underscores the value of in vitro models for cell‐cell interaction studies.^[^ ^164^, ^165^, ^166^ ^]^ Recently, by using acoustic bioprinting technology, Chen and colleagues obtained 3D microtissues that integrate PDOs and CAFs derived from the same patient, to investigate cancer invasion dynamics as well as response to treatment, by time‐lapse imaging.^[^ ^167^ ^]^

Furthermore, given the high versatility and reliability of these models, 3D co‐culture systems have been exploited also in high‐content phenotypic screenings and other applications as platforms for drug discovery initiatives (**Tables** **1**, **2**, **3**).^[^ ^168^ ^]^

**Table 1:** Schematic summary of technical approaches exploited for double cultures of CR‐cancer cells and CAFs.

Co‐culture settings					
Matrix	Device (methods)	Cell types	Application	Technology	Ref
DOUBLE CULTURES, Cancer cells and fibroblasts					
Collagen I	Transwell with mouse intestinal basal membrane. Lower compartment, fibroblast mixed in collagen; upper compartment, tumor cells	HT29, HCT116; patient derived CAFs and NFs	Invasion studies	IF; microscopy methods; time‐lapse imaging	[155]
BME; Matrigel	Transwell. Lower compartment, fibroblasts seeded directly or embedded in BME; upper compartment, organoids in matrigel droplets	Mouse and human CRC organoids; primary mouse colon fibroblasts	Tumor‐stroma interaction studies	qRT‐PCR analysis; contraction assay	[156]
Matrigel	Transwell. Lower compartment, fibroblasts; upper compartment, organoids in matrigel droplets	PDO and CAFs from surgical specimens of CRCs	Gene expression profiles	DNA microarray; viability assays	[165]
Collagen	Tumoroids in two layers of collagen gel	DLD‐1; human primary lung cancer‐derived fibroblasts	Invasion studies	Fluorescence imaging; IHC; time‐lapse experiments	[150]
	Tumoroids formed in agarose coated plates	SW480; MRC5	Drug response analysis; secretomes analysis	Fluorescence imaging; LC‐MS; viability assays	[157]
	Tumoroids formed in polydimethylsiloxane (PDMS) microwell platform.	HCT‐8; NIH3T3	Drug response analysis; co‐culture self‐organization; stiffness variation	Immunostaining; time‐laps imaging; mechanical stability measurement; WB analysis	[158]
	Tumoroids formed in hanging drop plates	HCT116; CCD‐18Co	High‐content drug discovery	Genomics screening; Imaging analysis; RNAseq	[168]
BME; Matrigel	Matrix droplet, Tumor cells and CAFs plated together. Transwell. Lower compartment, fibroblasts embedded in BME; upper compartment, organoids in matrigel droplets	PDO and CAFs from surgical specimens of CRCs	Tumor‐stroma interaction studies; drug response analysis	qRT‐PCR analysis; RNAseq; viability assays;	[163]
Collagen I	Matrix droplet into a force sensor	FET; human primary colorectal CAFs	Cell forces and tissue stiffness measurement	High‐resolution force sensor	[154]
Matrigel; Collagen I	Organoids plated in Matrix droplet and CAFs in suspension in the well	PDO and CAFs from surgical specimens of CRC liver metastasis	Tumor‐stroma interaction studies; mesenchymal‐like cancer model	scRNAseq; IF; live cell imaging; cell culture medium assays	[161]
hyaluronan‐gelatin hydrogels	CAFs seeded on top of each PDO Matrix droplet	PDO and CAFs from surgical specimens of CRCs	Drug response analysis; gene expression profiles	RNAseq; viability assays; immunostaining	[164]
Matrigel; Collagen I	Tumor cells and fibroblasts plated together in Matrix droplet or divided into individual layers in ALI culture.	PDO, NFs and CAFs from surgical specimens of CRCs	Tumor‐stroma interaction studies	Immunostaining; RNAseq; Proteomic; 3D migration assay; Cytokine array	[162]
GelMA	Bioprinting, PDO and CAF microtissue formed by acoustic droplets technology	PDO and CAFs from surgical specimens of CRCs	Migration and invasion models; drug response analysis	Time‐lapse imaging; IF	[167]

**Table 2:** Schematic summary of technical approaches exploited for double cultures of CR‐cancer cells and immune cells.

Co‐culture settings					
Matrix	Device (methods)	Cell types	Application	Technology	Ref
DOUBLE CULTURES, Cancer cells and immune cells					
Geltrex	PBMC + single cell‐dissociated organoids (20, 1 E, T ratio)	PDO and PBMC from surgical specimens of CRCs	Cancer‐T cells interaction studies	Flow cytometry; cytotoxicity assays	[170, 171]
BME; Matrigel	T cells + HT29 (1, 10 ratio); T cells + PDO	PDO from surgical specimens of healty colon or tumoral tissue; healthy donor PBMC (T cells); HT29; Colo205; Jurkat	Cancer‐T cells interaction studies; immune surveillance	Time‐lapse imaging, immunofluorescence	[172]
Matrigel	CD8 T cells + tumor cells (2, 1 or 5, 1 E, T ratios)	PDO from surgical specimens of CRCs; healthy donor PBMC (CD8^+^ Tcells)	Immunotherapy response analysis	Fluorescence imaging; flow cytometry	[173]
	Vδ2 T cells + CRC spheroids (1, 1 E, T ratio).	HCT15, SW620, DLD1 spheroids; healthy donors PBMC (Vδ2 T cells)	Immunotherapy response analysis	Viability and cytotoxicity assays; spheroid size measurement	[174]
	CD19^−^CD14^−^ sorted PBMCs or TILs + tumor cells (1, 1 E, T ratio)	HT‐29, DLD1; healthy donors CD19^−^CD14^−^ sorted PBMCs; patient‐derived tumor spheroids; autologous tumor‐infiltrating lymphocytes (TILs)	Immune infiltrate characterization; antitumor effects studies	Flow cytometry; IF; spheroid volume calculation	[175]
	CRC spheroids + human monocytes (1, 1 E, T ratio).	SW620‐CSC; healthy donors PBMC (monocytes)	Analysis of tumor hybrid cells properties	Migration assay; viability assay; flow cytometry; soluble immune‐checkpoint measurement	[177]
Matrigel	Organoids seeded on a Matrigel layer or on a layer of primary human colon fibroblasts + NK‐92 cells	PDO from surgical specimens of CRCs; normal PDO and primary fibroblasts from non‐pathological mucosa	CAR‐NK immunotherapy studies	Fluorescence imaging; viability and cytotoxicity assays	[179]

**Table 3:** Schematic summary of technical approaches exploited for organotypic multi‐cultures mimicking CRC.

Part I					
Co‐culture settings					
Matrix	Device (methods)	Cell types	Application	Technology	Ref
ORGANOTYPIC MULTI‐CULTURES					
	Tumoroids formed in agarose micro‐molds	HCT116; HIF; human monocytes	Drug response analysis, co‐culture self‐organization	Metabolic activity analyzers; size measurement; IHC; flow cytometry; live imaging	[183]
	Tumoroids, formed in cell‐repellent surface 96‐well microplates, co‐cultured with Immune cells	HT‐29; MS‐5; healthy donors PBMC (pan‐T cells and NK cells)	Immune infiltrate characterization; immunotherapy response analysis	Flow cytometry; IHC	[186]
Collagen I	CRC Spheroids seeded on the top of a blood endothelial cells layer covering collagen I‐embedded CAFs	SW480, SW620; HLFs;patient‐derived CAFs; BECs	Invasion model; drug response analysis	IF; qRT‐PCR; Circular chemo‐repellent‐induced defect (CCID) assay; Intracellular Ca2+ and 12(S)‐HETE assays	[188]
	Spheroids onto confluent monolayers of myofibroblasts, colon epithelial cells, or HUVEC	HT29, LS180, SW948; CCD‐18Co; CCD‐841CoTr; HUVECs	Drug response analysis; ROS detection	ELISA assay; ROS measurement	[184]
Matrigel	Mesenchymal and endothelial cells are co‐cultivated to obtain endothelial tubes (ETs). CRC tumor Spheroids are added after 5 days.	Patient derived‐tumor spheroids; bone marrow‐derived mesenchymal stromal cells (MSCs); HUVECs	Microvascular niche model	Time‐lapse imaging	[189]
Collagen I; Laminin	Matrix droplets, Cancer mass, formed mixing CRC cells with collagen I gel, are embebbed into a stromal compartment (HDFs+HUVECs).	HT29, HCT116; CCD‐841CoN; HDFs; HUVECs	Invasion studies; endothelial networks measurement	IF; optical projection tomography; qRT‐PCR	[187]
Collagen I; Laminin	Matrix droplets, Cancer mass, formed mixing CRC cells with collagen I gel, are embebbed into a stromal compartment (HDFs+HUVECs).	HT29, HCT116; HDFs; HUVECs	Invasion studies; endothelial networks interactions;	IF; qRT‐PCR; Collagen gel density measurement; WB analysis	[190]
Collagen I	ALI, The inner transwell containing tissue and collagen placed into an outer dish containing medium	Patient‐derived normal and tumoral tissue fragments	ALI PDO cultures characterization; drug response analysis	Immunostaining; cell viability assays.	[104]
Part II					
Collagen I	ALI, The inner transwell containing tumor tissue and collagen placed into an outer dish containing medium	Mouse and patient‐derived organoids	ALI PDO cultures characterization; immunotherapy response analysis	Exome sequencing; immunostaining; qRT‐PCR; cytotoxicity assays.	[105]
PCL scaffold; collagen I	3D bioprinting, cancer cells, CAFs, and TECs seeded onto sterile scaffolds	HCT116; activated HUVECs; HELFs	Co‐culture characterization, drug response analysis	IF analysis; RNAseq; qRT‐PCR; live/dead assays; in vivo experiments.	[107]
Rat‐derived dECM	CRC cell lines cultured on liver and lung dECM coated dishes	HT‐29, CRC119, SW480, and Caco2	Drug response analysis; histopathological analysis; gene expression profile analysis; metastatic model	Tissue decellularization; proliferation, apoptosis, anoikis and invasion assays; microarray; in vivo experiments.	[112]
Patient‐derived dECM scaffold	dECM injected with myofibrobastas, epithelial and endothelial cells	Human colonic organoids, myofibroblasts and microvascular endothelial cells	CRC progression model	Illumina sequencing; immunostaining;	[109]
Patient‐derived dECM scaffold; Collagen I	dECM injected with cancer cells	HT29, HCT116	Drug response analysis; EMT induction analysis; gene expression profile analysis; metastatic model	Tissue decellularization; IHC; IF; microarray, qRT‐PCR; migration assay; cytotoxicity assay.	[110, 111]
Fibrin gel	CoC, fibroblasts, endothelial and CRC cells injected into each tissue unit self‐organized within an extracellular matrix.	HCT116, SW480; NHLF; human endothelial colony‐forming cell‐derived endothelial cells	Vascularized micro‐tumor characterization; drug response analysis; tumor–stromal interactions.	IF; time lapse imaging; scRNAseq; gene expression profiles	[191]
Fibrin gel	CoC, the mixture of LFs, HUVECs and CRC cells injected into the central channel to form perfusable vassels. NK cells are introduced into the vessels through the side channels.	HT29, SW480, SW620, HCT116, LoVo and SW48; LFs; HUVECs; healthy donors PBMC (NK cells)	Extravasation, migration, immunotherapy response analysis	Time‐lapse imaging; immunocytochemistry	[192]
Matrigel; Collagen I; Collagen IV	CoC; epithelial channel (epithelial and cancerous cells) and endothelial channel separated by a porous membrane. CAFs, can be incorporated into the epithelial channel.	Caco2; HUVECs; HT29; HCT116; patient‐derived organoids and CAFs;	Intravasation; metabolic analysis; invasion;	IF; qRT‐PCR; invasion assays; LC‐MS	[122]
Thiolated hyaluronic acid, thiolated gelatin, and polyethylene glycol diacrylate (PEGDA)‐based hydrogel system	multi‐OoC, primary gut site (intestine epithelial cells and colon carcinoma cells) and secondary liver site in 2 chambers connected in series	HCT116; INT407; HepG2	Metastatic model; stiffness variation; drug response analysis.	IHC; migration‐invasion assays; cell viability assays	[124]
Thiolated hyaluronic acid, thiolated gelatin, and polyethylene glycol diacrylate (PEGDA)‐based hydrogel system	multi‐OoC, the main chamber populated by CRC cells connected with 4 other chambers (1.liver cells; 2.lung cells; 3.endothelial cells; 4.only matrix) by a double branching microfluidic channel.	HCT116; HepG2; A549; HUVECs	Metastatic model	Cell viability assays; fluorescent imaging.	[125]
Collagen I; Fibronectin	multi‐OoC, intestinal, hepatic, tumoral and connective culture chambers interconnected with microchannels on the microfluidic plate.	HCT116; HepaRG; Caco2; TIG121	Drug response analysis	Gene expression analysis; LC‐MS; cell viability assays;	[126]

#### 3.1.2 Cancer Cells and Immune Cells 3D Co‐Cultures

The lack of immune cell components in commonly used 3D systems restricts the comprehension of tumor cell interactions and their application to cancer immunotherapy. To overcome this limitation, several protocols have been established to isolate immune cell subtypes and explore their features in the tumor context, reproduce immune‐tumor cell interaction and set up novel therapeutic approaches. Although many 3D systems employ patient‐derived cells or cell lines, the use of animal cells offers a practical advantage in some cases to overcome limitations in accessing human samples. For example, studies involving immune cells derived from bone marrow, spleen, or other lymphoid organs might find using mice samples more advantageous due to easier accessibility.^[^ ^169^ ^]^

While many studies couple commercial CRC cell lines, grown as spheroids, with allogenic immune cells from healthy donors, autologous co‐cultures employing patient‐derived tumor cells and human peripheral blood mononuclear cells (PBMCs) have been recently introduced.

Dijkstra and colleagues developed a promising platform for preclinical evaluation of drug efficacy and resistance, especially for T‐cell‐based immunotherapy. After the isolation of immune cells (peripheral blood lymphocytes), tumor‐reactive T cells are expanded in the presence of interferon γ (IFNγ)‐stimulated‐autologous PDO and then evaluated for their reactivity and ability to kill tumor organoids. Similarly, Pesce and colleagues exploited a PDO‐CAF co‐culture to study cell interactions. They identify TMEM123 as a mediator of immune surveillance, controlling T‐cell migration, clustering and killing activity on cancer cells.^[^ ^170^, ^171^, ^172^ ^]^

To dissect the mechanisms of immunotherapy resistance in vitro, other studies employed allogeneic T cells in PDO co‐culture models.^[^ ^173^ ^]^ For example, the sensitivity of PDOs to cibisatamab (anti‐CEA/anti‐CD3 T cell bispecific monoclonal antibody) was assessed by co‐culturing GFP‐tagged PDO derived from multidrug‐resistant metastatic CRCs, with CD8 T cells isolated from healthy donor PBMCs. PDOs heterogeneity accurately reproduces patient complexity and therefore provides a reliable preclinical tool to predict response to therapy. Interestingly, CEA^high^ PDOs were highly sensitive to treatment with CD8 T cells and cibisatamab, whereas CEA^low^ cells were resistant to this treatment, demonstrating how heterogeneity and phenotypic plasticity in CEA expression could be a limitation in effective tumor targeting.^[^ ^173^ ^]^ Similarly, a co‐culture of spheroids derived from CRC established cell lines with Vδ2 T lymphocytes isolated from healthy donor PBMCs was exploited to demonstrate that zoledronate triggers Vδ2 T cells to kill and degrade CRC spheroids, while the anti‐EGFR monoclonal antibody cetuximab elicits the antibody‐dependent cellular cytotoxicity (ADCC) of tumor cells, exerted by effector lymphocytes.^[^ ^174^ ^]^

To examine the interactions between immune and tumor cells, an additional type of co‐culture employed CRC spheroids and CD19^−^CD14^−^ sorted healthy PBMCs. In this model allogeneic activated/memory T and NK cells can infiltrate tumor spheroids, leading to immune‐mediated tumor cell apoptosis and spheroid destruction, engaging both NKG2D‐MICA/B and NKG2A‐HLA‐E pathways. A combination of anti‐MICA/B and anti‐NKG2A antibodies showed a synergistic effect on immune‐mediated anti‐tumor response in this spheroid model. This was further confirmed using patient‐derived CRC spheroids and autologous TILs.^[^ ^175^ ^]^

Several studies have reported that tumor cell fusion phenomenon may be involved in metastasis. Cancer cells can fuse with neighboring cells (mesenchymal stromal cells – MSCs, fibroblasts, immune cells or other cancer cells) giving rise to tumor hybrid cells (THC) which exhibit high aggressiveness, drug resistance and ability to dodge immunity.^[^ ^176^ ^]^ Montalban‐Hernandez and colleagues obtained CD45^+^CD14^+^EpCAM^+^ THCs from fusion between CRC stem cells and human monocytes after 5 days of co‐culture, displaying enhanced metastatic abilities. Notably, the count of THC with this specific signature performed in CRC patients’ tumor tissue and bloodstream has a prognostic value predicting the development of distant metastases.^[^ ^177^ ^]^

Furthermore, the use of 3D co‐cultures allowed the development of preclinical platforms for CAR‐immune cells. Namely, the anti‐tumor efficacy of CAR‐engineered NK‐92 cells directed toward EpCAM and/or tumor‐specific antigens was evaluated on patient‐derived CRC organoids seeded on a Matrigel‐coated surface. Using luciferase‐GFP expressing organoids and CD45 staining of NK cells, CAR‐NK cytolytic activity was monitored by quantitative viability assay and confocal live‐imaging at a single‐organoid level.^[^ ^178^, ^179^ ^]^ Considering the specific architecture and cellular microenvironment that CAR‐T cells encounter especially in solid tumors, increasing studies have been developed for CAR‐T cells in 3D models, using low‐adhesion plates, scaffolds or hanging drop systems.^[^ ^180^, ^181^, ^182^ ^]^

Given the increasing interest in immune checkpoint inhibitors, CAR T/NK cells, and patient‐specific treatments, 3D co‐culture approaches may provide a valuable tool to improve cancer immunotherapies and overcome the immunosuppressive TME of CRC.

### 3.2 Organotypic Multi‐Cultures

While the simplification of TME is necessary to dissect the communication of cancer cells with specific cell types, the efforts to recapitulate in vitro the complexity of a given tissue are of paramount importance to obtain preclinical models able to reproduce tumor behaviour, to predict the response to therapy and replace/reduce the use of mouse models. The technical systems described above were utilized to generate co‐cultures involving multiple cell types, with the aim of developing organotypic multi‐cultures that facilitate the study of tumor evolution, as immune escape, the extravasation process, and migration. Here, we will describe few examples of successful multiple co‐cultures from the most recent and relevant literature on CRC.

A 3D model of CRC multicellular tumor spheroids (MCTS) combined epithelial colon cancer cells (HCT116), human intestinal fibroblasts and human monocytes, seeded in agarose micro‐molds. Self‐aggregated cells underwent spatial organization, with fibroblasts localized in the core, surrounded by epithelial cells and macrophages. Of note, some macrophages penetrated inside the MCTS, resembling CD68^+^ macrophages usually located in the tumor invasive front area. This 3D structure mimics some cancer features such as fibronectin deposition (directly correlated with the presence of fibroblasts), a necrotic core formation and the ability to polarize monocytes to pro‐tumor M2‐like macrophages.^[^ ^183^ ^]^

Another model tried to reproduce CRC progression in vitro, mimicking the early contact of cancer cells with the colonic epithelium and myofibroblasts, followed by the interaction with the endothelium during the metastatic dissemination. To this end, Paduch and colleagues developed a co‐culture system of human CRC spheroids (HT29, LS180, SW948) representing different tumor grades, with human normal colon epithelial, myofibroblast, and endothelial cells. The adhesion of tumor spheroids to colon epithelium and myofibroblast monolayers modulated the proinflammatory cytokines IL‐1 beta, IL‐6, TNF‐alpha and radicals (ROS and NO) levels in a tumor grade‐dependent manner, with a significant increase of IL‐6 production in advanced CRC. The addition of cytotoxic drugs (5‐FU, leucovorin and camptothecin) influenced the culture milieu, changing the levels of radicals and cytokines, decreasing IL‐6 and increasing TNF‐α and ROS production.^[^ ^184^ ^]^

Different strategies, including 3D systems, have been exploited to turn the immunosuppressive TME into an immunogenic environment. The chemokine CXCL12 (SDF‐1), mainly expressed by CAF in the TME, has been associated with checkpoint inhibitors resistance through T‐cell exclusion.^[^ ^185^ ^]^ Zboralski and colleagues developed a spheroid system consisting of CXCL12‐secreting MS‐5 stromal cells and cancer cell lines from four different origins (pancreatic adenocarcinoma, CRC, non‐small cell lung cancer – NSCLC and glioblastoma cells). The addition of immune cells (PBMCs or isolated primary human pan T cells) to the heterotypic spheroids demonstrated that CXCL12 inhibitor NOX‐A12 enhanced infiltration of CD8^+^ T cells, CD4^+^ T cells, NK cells, and, to a lesser extent, Treg and B cells into the tumor‐stroma spheroids. NOX‐A12 synergized with anti–PD‐1 checkpoint inhibition both in vitro and in a syngeneic murine model of CRC, significantly reducing tumor volumes, suggesting a possible strategy to overcome the resistance to anti–PD‐1 treatment.^[^ ^186^ ^]^

Recent approaches mimicking vascular microenvironment include organoids cultured in tissue‐engineered vascular models and organ‐on‐chips. A 3D CRC model of vascularized stroma is based on the use of a high‐density type I monomeric collagen containing a central cancer mass embedded into a stromal compartment of endothelial cells and fibroblasts (tumoroids). Comparing two colorectal cancer cell lines (HT29 and HCT116), the authors reported that HCT116 cells were more invasive than HT29. Furthermore, tumoroids containing highly invasive HCT116 cancer masses formed less branched vascular networks, resembling “leaky” vasculature of highly metastatic tumors.^[^ ^187^ ^]^

Another 3D model of CRC invasion into the stroma was established using CRC spheroids seeded on the top of a blood endothelial cells (BECs) layer covering collagen I‐embedded CAFs. In this system, tumor cells secreted the “‘endothelial retraction factor”’ 12(S)‐HETE, a product of lipid metabolism which perturbs intercellular junction, causing the retraction of CAFs and blood endothelial barriers. These entry gates may facilitate cancer cell invasion and metastatic spread, and therefore the therapeutic use of calcium inhibitors was suggested to interfere with 12(S)‐HETE signalling and cell retraction.^[^ ^188^ ^]^

In a study on the role of perivascular bone marrow as a reservoir of disseminated tumor cells (dTCs), Möhrmann and colleagues generated an organotypic microvascular co‐culture model. In this system, CRC tumor spheroids were added as single cells to the co‐colture, 5 days after seeding HUVECs and MSCs, showing that tumor cells migrate toward, localize and proliferate close to endothelial tubes.^[^ ^189^ ^]^

To closely reproduce all the fundamental cues of TME in vitro, it appears crucial to reconstruct cellular subset interactions together with the ECM‐specific features. As already discussed, ECM density influences proliferation, invasion and vascularization. To mimic the solid nature of tumor mass, ECM density can be modulated by a strategy of plastic compression. As recently reported, it is possible to obtain an artificial cancer mass (ACM) of collagen hydrogel containing CRC cell lines, supplemented with EC (HUVEC), dermal fibroblasts, laminin and further compressed. In this system, ECM density and laminin were both proven as critical factors regulating cancer cell invasion and vascular network formation.^[^ ^190^ ^]^ Interestingly, a number of recent studies based on microfluidic approaches attempted to reproduce the complex 3D TME through the control of fluid volumes, cell compartmentalization, paracrine signals and microvasculature, mimicking different features of TME such as cells‐crosstalk, angiogenesis, molecular gradients, microbiome or drug response. Devices using organ‐on‐a‐chip technology allowed to recreate angiogenic microvascular networks, deliver drugs or nanoparticles and study therapy responses.^[^ ^117^, ^121^, ^191^ ^]^ Song J. and colleagues developed a 3D tumor vasculature system on a high‐throughput microfluidic platform that incorporates CRC cells and fibroblasts around perfusable vascular networks (HUVEC); adding NK cells into the vascularized network induced their extravasation, migration and immune‐cell‐mediated cytotoxicity.^[^ ^192^ ^]^

These examples show how the application of novel technologies to in vitro modelling of cancer dramatically broadens the potential of these approaches. Further, the integration of different primary cells makes this expanding research field extremely exciting and promising.

## 4 Preclinical Applications of CRC In Vitro Models

To date, 2D cancer cell cultures have been widely used for drug sensitivity testing and identifying potential therapeutic compounds for patient treatment. However, as mentioned before, 2D systems lack multiple features of the original tumor and the results often do not directly translate to clinical settings. In contrast, 3D tumor models offer an ideal platform for high‐throughput drug screening and for toxicity and efficacy studies, enabling the development of personalized therapies and prediction of patient responses to treatment.^[^ ^193^ ^]^ Although not all 3D models are suitable for high‐throughput screening due to their intrinsic complexity or lack of long‐term stability, organoids have emerged as a promising tool for *ex vivo* drug testing. Patient‐derived organoids (PDOs) capture phenotypic and genotypic features of the patient's tumor, allowing for extensive and rapid *ex vivo* drug testing. For example, Vlachogiannis and colleagues generated a biobank of PDOs from metastatic, heavily‐pretreated colorectal and gastroesophageal cancer patients, to evaluate molecular profiles and drug responses. PDOs were established from sequential biopsies (baseline, at the time of best response, disease progression), and multi‐region sites. 19 PDOs were exploited in a 3D screening using a library of 55 drugs in phase I‐III clinical trials or in clinical practice. By comparing PDO drug responses and PDO‐based orthotopic mouse tumor xenograft with patients’ responses in clinical trials, they conclude that PDOs can recapitulate patient's heterogeneity and serve as a valuable tool for improving specific therapies.^[^ ^194^ ^]^ The integration of PDXs data with whole exome sequencing (WES) analysis and high‐throughput drug screening on PDO can reveal specific drug sensitivities and uncover new therapeutic approaches for CRC.^[^ ^195^ ^]^ A similar approach was used to analyze 20 CRC patients’ organoids with WES and high‐throughput screening of a panel of 83 drugs.^[^ ^94^ ^]^ The study demonstrated that KRAS‐mutant organoids were resistant to the anti‐EGFR inhibitors (cetuximab and afatinib), while RNF43‐mutant organoids were sensitive to WNT secretion inhibitors and TP53 loss of function mutations were associated with resistance to nutlin‐3a (MDM2 inhibitor). Similarly, Kondo and colleagues performed a larger scale screening using a panel of 2427 compounds on CRC organoids to identify several “hit” drugs.^[^ ^196^ ^]^

To overcome the time‐consuming process of PDO derivation and expansion, Shen's lab developed an automatic microfluidics droplet platform capable of generating patient‐derived micro‐organospheres (MOS), for clinical high‐throughput drug selection within an extremely short timeframe. By analysing samples from eight metastatic CRC patients, the authors showed that MOS can provide results within 7–14 days from obtaining a biopsy, with drug responses correlating with clinical outcomes (119 different FDA – approved small molecule inhibitors tested). Importantly, MOS preserved stromal and immune cells of the original tumor, thus representing a powerful tool for immunotherapy testing.^[^ ^197^ ^]^

Moreover, the integration of gut microbiome in the 3D co‐culture systems leads to a better understanding of the microbial contribution to CRC development, identifying CRC‐associated bacteria across the different tumor stages. Based on these findings, possible approaches to improve cancer treatment and prevention arise, with the potential to harness the anti‐tumor effects of metabolites produced by specific gut bacteria.^[^ ^198^ ^]^ For example, Greenhalgh et al used a microfluidics‐based human‐microbial co‐culture system called HuMiX to analyze diet‐host‐microbe molecular interactions. They studied the effects of a simulated high‐fiber diet (medium containing major non‐digestible carbohydrates and additional prebiotics including arabinogalactan, xylan, and soy), integrated with the probiotic *Lactobacillus rhamnosus* Gorbach‐Goldin (LGG) and human CRC cells (Caco‐2) co‐culture. Of note, the symbiotic regimen caused the downregulation of genes involved in protumoral pathways and drug resistance and represents a promising tool for developing personalized dietary treatments for CRC patients.^[^ ^199^ ^]^

These expanding fields may yield solid pre‐clinical tools that will add huge opportunities for both drug discovery and personalized treatment.

## 5 Conclusions and Perspectives

In conclusion, 3D systems provide the opportunity to develop sophisticated “patient avatars”, personalized patient‐derived preclinical models that reproduce the response to specific therapeutic regimens, allow to generate integrated multiple “omics” data and to perform high‐throughput screenings for drugs discovery.

The integration of sophisticated 3D platforms and automation systems enhances experimental efficiency and accuracy. Moreover, incorporating quantitative methods provides deeper insight into cellular behaviour, interactions, and treatment responses within the microenvironment. The quantitative analysis encompasses different techniques such as cell live/dead staining, migration and invasion assays, high‐content imaging, gene/protein expression profile, and spatial transcriptomics. Additionally, the development of software and machine learning tools further facilitates data interpretation and extraction of meaningful insights from complex datasets, thereby advancing our understanding of cellular behaviour within 3D systems. These avatars might be exploited to predict the response to therapy, with the final aim to identify the most effective treatment for individual patients or patient subsets, while minimizing off‐target toxicities.^[^ ^200^ ^]^

The increased complexity of some avatars allows for a better reproduction of tumor behavior, especially in drug response studies. On the other hand, such a complexity does not guarantee a proper resolution in the detailed analysis of specific components of CRC TME. Indeed, simpler models are more prone to the dissection of crosstalk mediators and interactions. We believe that a combined use of all the technologies available may be the best strategy to understand the contribution of TME and to generate solid pre‐clinical models that will stand as valid alternatives to animal use.

## Conflict of Interest

The authors declare no conflict of interest.
